# Dissolving microneedle array patches containing mesoporous silica nanoparticles of different pore sizes as a tunable sustained release platform

**DOI:** 10.1016/j.ijpharm.2024.125064

**Published:** 2025-01-25

**Authors:** Juan L. Paris, Lalitkumar K. Vora, Ana M. Pérez-Moreno, María del Carmen Martín-Astorga, Yara A. Naser, Qonita Kurnia Anjani, José Antonio Cañas, María José Torres, Cristobalina Mayorga, Ryan F. Donnelly

**Affiliations:** a Allergy Research Group, Instituto de Investigación Biomédica de Málaga y Plataforma en Nanomedicina-IBIMA Plataforma BIONAND. RICORS “Enfermedades inflamatorias”, Málaga, Spain; b Universidad de Málaga, Málaga, Spain; c School of Pharmacy, Queen’s University Belfast, Medical Biology Centre, Belfast, UK; d Allergy Unit, Hospital Regional Universitario de Málaga-HRUM, Málaga, Spain

**Keywords:** Microneedles, Mesoporous silica nanoparticles, Drug delivery, Nanomedicine

## Abstract

Dissolving microneedle array patches (DMAP) enable efficient and painless delivery of therapeutic molecules across the *stratum corneum* and into the upper layers of the skin. Furthermore, this delivery strategy can be combined with the sustained release of nanoparticles to enhance the therapeutic potential in a wide variety of pathological scenarios. Among the different types of nanoparticles that can be included in microneedle formulations, mesoporous silica nanoparticles (MSN) of tunable pore sizes constitute a promising tool as drug delivery systems for cargos of a wide range of molecular weights. In this work, a new preparation method was developed to produce DMAP containing *ca*. 2.3 mg of MSN of different pore sizes located mainly in the microneedle tips. The successful insertion of these DMAPs was confirmed *in vitro* (using Parafilm), *ex vivo* (using excised neonatal porcine skin) and *in vivo* (in the back of mice). The dissolution of the microneedles and deposition of the nanoparticles inside the skin were also confirmed both *ex vivo* and *in vivo* using fluorescent nanoparticles (with an intradermal deposition of 20.9 ± 7.26 % of the MSN in each DMAP in neonatal porcine skin). Finally, the *in vivo* release of the cargo from nanoparticles deposited inside mouse skin after microneedle insertion was confirmed through *in vivo* fluorescence measurements.

## Graphical abstract

**Figure:**
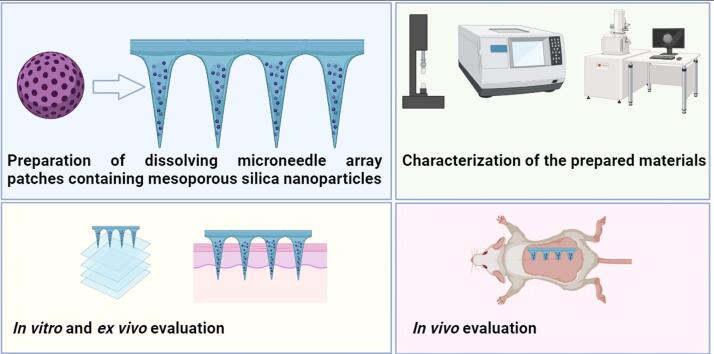

## 1 Introduction

Dissolving microneedle array patches (DMAPs) are arrays of needle-like structures with microscale diameters and lengths up to 1 mm mainly made using polymers that dissolve with the interstitial fluid after their insertion into the skin (Lee et al., 2008, Priya et al., 2023, Sartawi et al., 2022). Given their small size, when they are inserted in the skin, while they enable the deposition of drugs inside the upper layers of the skin (and across the external barrier of *the stratum corneum*), they do not reach blood vessels or pain receptors, resulting in no bleeding or pain. For this reason, they are often proposed as an alternative drug administration option without the need for conventional injections (Amani et al., 2021, Cheung and Das, 2014, Yang et al., 2021). DMAPs have been proposed for a wide variety of therapeutic applications, such as vaccines (Chandrasekharan et al., 2019, Mistilis et al., 2017, Vassilieva et al., 2015), cancer treatment(Pei et al., 2018, Zhao et al., 2018), migraines (Tas et al., 2017), fungal infections (Peng et al., 2021), psoriasis (Du et al., 2019) or malaria(Volpe-Zanutto et al., 2021), among many others. Within the polymer matrix that makes up the microneedles, different micro- or nano-particle based formulations can be included to improve the therapeutic performance of the formulation (Larrañeta et al., 2016). For example, if nanoparticles with sustained drug release are administered through DMAP, their deposition inside the skin creates a reservoir of the drug at the site of administration, which is slowly released from the nanoparticles, reducing the need for continuous re-administration of the treatment. Based on this concept, DMAP has been prepared containing many types of nanoparticles, such as liposomes (Guo et al., 2013, Qiu et al., 2016, Zhou et al., 2021), cubosomes (Ramalheiro et al., 2020, Rattanapak et al., 2013), polymeric nanoparticles(Hu et al., 2022, Mönkäre et al., 2018, Permana et al., 2020), metallic nanoparticles (Permana et al., 2021) and mesoporous silica nanoparticles (MSNs) (Zhang et al., 2023). Among these different types of nanoparticles, MSNs with tunable pore sizes (generally in the range of 2–20 nm in diameter (Paris et al., 2023)) constitute a particularly promising tool as drug delivery systems(Durfee et al., 2016, Hong et al., 2020, Paris et al., 2023), as they present a large loading capacity for cargos of a wide range of molecular weights(Baeza et al., 2015, Vallet-Regí et al., 2018, Zhou et al., 2018). MSNs have been proven to be safe and to undergo dissolution in physiological environments, giving rise to nontoxic degradation products such as silicic acid, which can be safely excreted in urine(Paris et al., 2019, Paris et al., 2017). Several microneedle arrays (either dissolving or nondissolving) containing MSNs have been previously reported (Du et al., 2018, Wang et al., 2021, Xu et al., 2017, Zhang et al., 2023). However, the amount of MSNs contained in previously reported microneedle formulations for intradermal deposition of the nanoparticles was relatively low, between 9.7 µg per array for coated microneedles (Du et al., 2018) and 362 µg per array for dissolving microneedles (Du et al., 2024), and would likely not be enough to deliver a therapeutically relevant dose of drug for most potential applications. Furthermore, the possibility of preparing DMAP with MSNs of different pore sizes has not previously been reported to the best of our knowledge. The development of an efficient method to prepare DMAP containing large quantities of MSNs of different pore sizes located specifically at the microneedle tips would provide a tunable drug delivery platform. This platform could be adapted for many different therapeutic applications, as the pore size of the nanoparticles could be tailored for the desired drug (Hong et al., 2020), and might even allow for combination therapies in which a mixture of different MSN formulations could be codelivered through a single DMAP. In this work we report for the first time a simple method to obtain DMAP containing large amounts of MSNs of tunable mesopore sizes using nanoparticles in powder form. Different nanoparticle-containing DMAP formulations were prepared and characterized, and their therapeutic potential was assessed through a variety of *in vitro, ex vivo* and *in vivo* methods. The modular platform presented here could be adapted to deliver sustained release formulations of therapeutic molecules over a wide range of molecular weights, either as monotherapies or as combination therapies with multiple drugs.

## 2 Materials and methods

### 2.1 Materials

The following reagents were purchased from Merck (Sigma–Aldrich, Spain) and were used without further purification: tetraethylorthosilicate (TEOS), cyclohexane, triethanolamine, cetyltrimethylammonium chloride (CTAC), ammonium nitrate, ethanol, hydrochloric acid, fluorescein isothiocyanate (FITC), rhodamine B isothiocyanate (RITC), aminopropyltriethoxysilane (APTES), fluorescein sodium salt, insulin, ovalbumin (OVA), phosphate buffered saline (PBS) tablets, polyvinyl alcohol 9–10 kDa (PVA), polyvinyl pyrrolidone K 30 (PVP, 40 kDa) and agarose.

### 2.2 Synthesis of MSNs

MSNs of different pore sizes were prepared by a previously described biphasic method based on the condensation of TEOS in a biphasic water/cyclohexane system, using triethanolamine as the base and CTAC as the structure-directing agent surfactant(Durfee et al., 2016, Paris et al., 2023). The aqueous phase was composed of a mixture of 24 mL of a commercial aqueous solution of CTAC (25 % w/v)), 0.18 g of triethanolamine and 36 mL of deionized water. The organic phase consisted of 20 mL of a mixture of cyclohexane with TEOS. The concentration of TEOS depended on the material to be prepared: 40 % for small-pore MSN (S-MSN), 20 % for medium-pore MSN (M−MSN), 10 % for large-pore MSN (L-MSN) and 5 % for extra-large pore MSN (XL-MSN). The synthesis reaction was carried out at 50 °C for 24 h. Then, the surfactant was extracted by ion exchange with an ethanolic solution of ammonium nitrate (10 mg/mL) at reflux for 1 h, followed by a second reflux for 2 h in an ethanolic solution of 12 mM HCl. Finally, the material was washed with ethanol 3 times to obtain the desired materials, which were dried and stored at room temperature until further use. Fluorescent MSN were also obtained by adding a mixture of 1.5 mg of fluorescein FITC or RITC and 15 µL of APTES in 1 mL of ethanol in the aqueous phase during MSN synthesis.

### 2.3 Cargo loading and release from MSNs

Fluorescein sodium salt (as a model for small molecule drugs), insulin (as a model for therapeutic peptides) or OVA (as a model for therapeutic proteins) was loaded in MSNs by dispersing 10 mg of MSNs in a 10 mg/mL (fluorescein sodium salt, OVA) or in a 2.5 mg/mL (insulin) solution of the cargo in PBS (10 mM, pH = 7.4) and stirring overnight. Then, the loaded particles were collected by centrifugation, and the nonloaded cargo was quantified from the supernatant by UV–Vis spectrophotometry. For release experiments, loaded particles were suspended in PBS and stirred at 37 °C. At different time points, the particles were centrifuged, released cargo was quantified by fluorimetry (fluorescein sodium salt, λ_EX_ = 580 nm; λ_EM_ = 520 nm) or UV–Vis spectrophotometry (insulin, λ_ABS_ = 274 nm; OVA, λ_ABS_ = 280 nm), and the particles were resuspended in fresh PBS to continue stirring at 37 °C.

### 2.4 Characterization techniques

Dynamic light scattering (DLS) and Z-potential measurements were performed with a Malvern Zetasizer Nano ZS90 instrument, checking both particle size and surface charge. The instrument used was equipped with a “red laser” (ʎ = 300 nm), and DLS measurements were performed with a detection angle of 90°, while the Smoluchowski approximation was used for Z-potential measurements. For Fourier transform infrared spectroscopy (FTIR), a Jasco 4100 FT/IR machine equipped with an attenuated total reflectance (ATR) accessory was used. To check the morphology and the different pore sizes of the nanoparticles, the characterization of the nanoparticles was performed by transmission electron microscopy (TEM) on a Thermo Fisher Scientific Tecnai G2 20 Twin using copper grids of mesh size 200 coated with a Formvar-Carbon film. Scanning electron microscopy (SEM) was carried out on an FEI Quanta-250 microscope (Thermo Fisher Scientific, USA) after coating the samples with a thin layer of gold under vacuum. Nitrogen adsorption (in a Micromeritics ASAP 2020 unit) measurements were carried out at the Central Research Support Services (SCAI) of the University of Malaga (UMA). The pore width of the different MSN formulations was determined from the maximum Barrett-Joyner-Halenda (BJH) Adsorption Cumulative Pore Volume distribution *vs* pore width graph obtained by N_2_ adsorption. Fluorimetry and UV–Vis spectrophotometry were carried out using a plate reader (FLUOstar Omega Microplate Reader, BMG LABTECH, Germany). DMAPs were visualized and imaged using a stereomicroscope (Leica EZ4 D, Leica Microsystems, Milton Keynes, UK). Constant compressive force was applied through a TA-TX2 Texture Analyzer (Stable Microsystems, UK). Optical coherence tomography (OCT) was carried out in an EX-101 device (Michelson Diagnostics Ltd., Kent, UK). Fluorescence microscopy was carried out on an EVOS FL microscope (Thermo Fisher Scientific, USA). Two-photon fluorescence microscopy was carried out in a Leica TCS SP8-MP multiphoton excited fluorescence upright microscope (Leica Microsystems, UK). *In vivo* fluorescence was evaluated with In-Vivo Xtreme equipment (Bruker, Germany).

### 2.5 Preparation of MSN-loaded DMAP

MSN-loaded DMAP was prepared using a negative silicone mold with a design containing 600 pyramidal microneedles (750 µm in length) through the following procedure: i) the microneedle tip region was filled with MSNs in powder form using a spatula, passing the powder over the microneedle region of the molds several times with gentle pressure until no more powderwas introduced and excess powder was then removed; ii) a 20 % (w/w) polymer solution (PVA and PVP at a 1:1 wt ratio) in deionized water was added to each mold, followed by centrifugation and removal of excess polymer solution; iii) 800 µL of a 40 % (w/w) polymer solution (PVA and PVP at a 1:1 wt ratio) in deionized water was added to each mold, followed by centrifugation; iv) the samples were left to dry for 24 h at room temperature and for 24 additional hours at 37 °C. Then, the DMAP was removed from the mold and stored until further use. Control DMAPs without MSNs were prepared by a similar procedure, skipping steps i) and ii).

### 2.6 *In vitro* evaluation of DMAP insertion and dissolution

First, DMAP insertion was evaluated *in vitro* using a Parafilm M® insertion model(Larrañeta et al., 2014) by applying a compressive force of the DMAP against 8 layers of Parafilm M® for 30 s, either 32 N using a Texture Analyser or manually using thumb pressure (32 N was previously selected as a comparable force to the force a human produces when applying thumb pressure on microneedles (Abdelghany et al., 2019, Larrañeta et al., 2014)). The depth of insertion was then evaluated by examining the different Parafilm M® layers under a stereomicroscope. DMAP dissolution was then evaluated by introducing DMAP in PBS and imaging the microneedles at different time points using a stereomicroscope. Finally, DMAP insertion and dissolution were also evaluated in a 3 % agarose gel. Five minutes after insertion in the agarose gel, the baseplate of the DMAP was removed, and the fate of FITC-labelled or fluorescein sodium-loaded MSNs was evaluated 1 h later (after incubation at 37 °C) using fluorescence microscopy.

### 2.7 *Ex vivo* experiments using neonatal porcine skin

Full thickness neonatal porcine skin was used as a skin model, with samples obtained from stillborn piglets and immediately (<24 h after birth) excised. Skin samples were shaved and stored in sealed Petri dishes at − 20 °C until use. Prior to use, skin samples were equilibrated in PBS. Insertion of DMAP into neonatal porcine skin was carried out as described for the Parafilm M® *in vitro* model. Insertion was evaluated by OCT, and dissolution was evaluated after different time points at 37 °C. During DMAP *in situ* dissolution, samples were kept in a sealed container where PBS-wetted paper was placed below the skin samples to prevent them from drying. Quantification of deposited fluorescent MSNs was carried out by fluorimetry following the extraction of the MSNs from excised skin into PBS by thorough sonication in an ultrasound bath. The diffusion of FITC-labelled MSNs or fluorescein sodium (from loaded MSNs) from DMAP across neonatal porcine skin was evaluated using Franz diffusion cells (Crown Glass Co. Inc., Sommerville, USA). Receptor compartments were filled with PBS, and the temperature was controlled during the experiment at 37 °C. Skin samples were secured to the donor compartment of the diffusion cell using cyanoacrylate glue with the *stratum corneum* side facing the donor compartment. DMAPs were inserted as previously described into the center of the skin sample. DMAPs were kept in place during the experiment by a cylindrical metal weight (diameter 11 mm, mass 11.5 g) on their upper surface. With DMAP in place, donor compartments were mounted onto the receptor compartments of the Franz cells. Using a long needle, 0.2 mL of sample was removed from the receptor compartment at defined time intervals and replaced with an equal volume of PBS. Sink conditions were maintained throughout the experiment. The concentrations of FITC-labelled MSNs or fluorescein sodium in the receiver medium were determined by fluorimetry.

### 2.8 *In vivo* experiments in mice

Mouse studies were carried out following Spanish national and European regulations (RD1201/2005, 32/2007, 2010/63/UE and RD53/2013). The mice were hosted at IBIMA-Plataforma BIONAND (Registration No. ES 290670001687). All procedures followed the 3R principles and received appropriate regulatory approval before starting (protocol 18/11/2021/180 approved by both the Institutional Ethics Committee and by *Consejería de Agricultura, Ganadería, Pesca y Desarrollo Sostenible, Junta de Andalucía*). The mice were anaesthetized during the different procedures and finally sacrificed by cervical dislocation. After obtaining the corresponding samples, the mice were stored and incinerated according to institutional guidelines.

To evaluate DMAP insertion and MSN deposition in mice, DMAP loaded with FITC-labelled M−MSNs was used. Additionally, DMAP containing fluorescein (sodium salt)-loaded M−MSN were also used to evaluate *in vivo* cargo release. Five- to six-week-old BALB/c mice (both male and female, n = 3 mice per group) from Janvier Lab (Saint-Berthevin Cedex, France) were used. For DMAP administration, back hair was first removed from the mice by using a hair-removal cream under intraperitoneal anaesthesia (xylazine + ketamine mixture). After washing the skin with saline solution to remove the cream and gently drying it with paper, the DMAP was inserted on the back skin by applying appropriate pressure for 30–60 s with the mice still under anaesthesia, and then adhesive tape was used to fix the DMAP in the same position. After 2 h, the baseplates were removed, and excess polymer on the skin surface (not inserted) was removed with PBS. After different time points, nanoparticle fluorescence in the mice was examined using an *in vivo* imaging system. At the endpoint (3 days after DMAP administration), the mice were euthanized by cervical dislocation under anaesthesia, and the skin was observed by fluorescence microscopy. The deposited MSN amount was quantified as described for the *ex vivo* experiments.

## 3 Results and discussion

### 3.1 Synthesis and characterization of MSNs

**Fig. 1:**
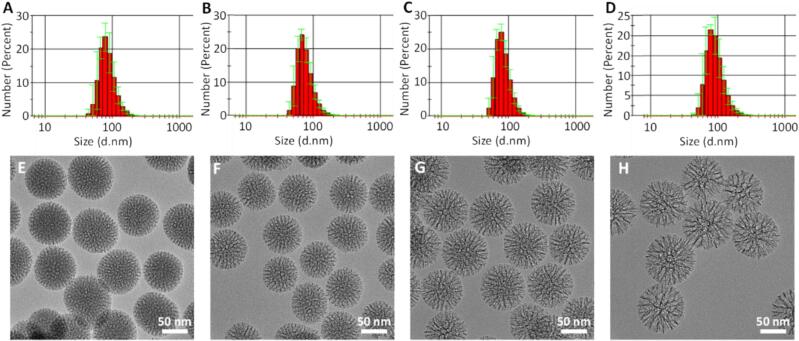
DLS size distribution histograms of MSNs: S-MSN (A), M−MSN (B), L-MSN (C) and XL-MSN (D); TEM micrographs of MSNs: S-MSN (E), M−MSN (F), L-MSN (G) and XL-MSN (H).

MSNs with different pore sizes (from smaller to larger: S-MSN, M−MSN, L-MSN and XL-MSN) were prepared and characterized. The successful removal of the surfactant was confirmed by FTIR spectroscopy (Figure S1). The size histograms obtained by DLS show peak particle diameters of approximately 100 nm (Fig. 1**A-D**), with Z average values in the range of 110–160 nm and narrow size distributions (polydispersity index, PDI, below 0.2) for all the obtained particles (Table 1). The nanoparticle surface charge was negative for all the prepared MSNs, as would be expected by the presence of silanol groups on the external surface of silica nanoparticles (Table 1). The round morphology and porosity of the MSNs could also be observed in the TEM micrographs of the prepared nanoparticles (Fig. 1**E-H**).

**Table 1:** Characterization of the prepared MSNs by DLS, Z potential and N_2_ adsorption.

	S-MSN	M−MSN	L-MSN	XL-MSN
Hydrodynamic diameter (Z Average, nm)	123.5 ± 2.33	130.2 ± 0.89	117.1 ± 2.39	155.4 ± 2.08
PDI	0.15 ± 0.02	0.19 ± 0.01	0.12 ± 0.03	0.16 ± 0.03
Z Potential (mV)	−8.31 ± 0.7	−24.5 ± 0.2	−12.1 ± 0.4	–32.8 ± 0.4
BET* Surface Area (m^2^/g)	321.9 ± 24.6	522.8 ± 148.9	560.9 ± 149.3	679.4 ± 11.1
Pore volume (cm^3^/g)	0.39 ± 0.02	0.71 ± 0.21	0.93 ± 0.18	1.38 ± 0.05
Pore width (nm)	3.33 ± 0.21	5.65 ± 0.14	8.46 ± 0.09	11.77 ± 0.64

* Brunauer–Emmett–Teller (BET) method.

The textural properties of the prepared MSNs were further confirmed by N_2_ adsorption (Table 1**,** Figure S2). All the materials presented large surface areas (in the range of 300–700 m^2^/g), which are typical of mesoporous silica materials. The pore diameters obtained by N_2_ adsorption confirmed the successful preparation of MSNs with 4 different pore sizes, in the range of 3 to 12 nm (S-MSN < M−MSN < L-MSN < XL-MSN). These results were in good agreement with the characteristics of MSNs prepared by other authors using the same method(Durfee et al., 2016).

**Fig. 2:**
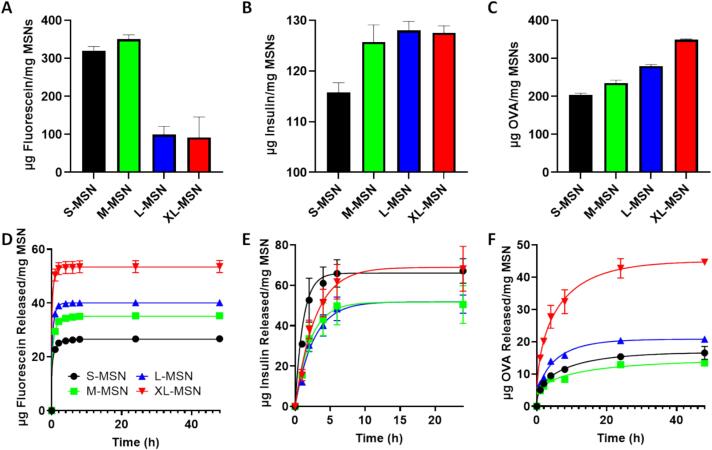
Cargo loading and release experiments in/from MSNs of different pore sizes. Loading of fluorescein sodium salt (A), insulin (B) and OVA (C); Release of fluorescein sodium salt (D), insulin (E) and OVA (F). Data are means ± SDs, n = 3.

The effect of pore size on cargo loading and release was evaluated using fluorescein sodium salt as a model for small molecule drugs, insulin as a model for therapeutic peptides and OVA as a model for therapeutic proteins and macromolecules (Fig. 2, Table S1). The loading of OVA reached a maximum for XL-MSN and decreased as pore size was reduced. This result was in good agreement with previous reports that have also shown that MSNs with extra-large pore sizes presented increased OVA loading compared to particles with smaller pores(Hong et al., 2020). On the other hand, fluorescein sodium salt loading was maximum for particles with small or medium pores (S-MSN and M−MSN) and drastically decreased for particles with larger pores. Finally, insulin loading was similar among all MSN materials with the exception of S-MSN, which presented lower loading. These data indicate that to obtain optimal cargo loading in MSNs, the pores should be large enough to accommodate the cargo, but if the pore size is too large, the loading efficiency decreases. Thus, the pore size should be tuned depending on the molecular weight of the cargo, requiring smaller pores for small molecules and larger pores for macromolecules such as proteins. Despite this, when cargo release experiments were carried out, larger pore particles generally presented faster release kinetics for different model cargos, as the larger pores provide easier accessibility to the solvent, which drives release. This result is also in good agreement with previous reports(Hong et al., 2020, Pérez-Moreno et al., 2024). However, this general trend did not accurately describe cargo release under 2 conditions: release of insulin and OVA from S-MSN particles. In both cases, S-MSN release was faster than expected, above that of M−MSN in the case of OVA and above that of all other MSN in the case of insulin. In these cases, the pore size of S-MSN particles is too small to host these cargos, and therefore the majority of the cargo will be adsorbed on the external particle surface, which leads to faster release. In any case, our results indicate that the textural properties of MSN should be tailored to specific therapeutic cargos to optimize both their loading and release behaviour. Furthermore, taking these results into account, a combination therapy scheme could be envisioned where drugs of different molecular weights can be co-administered in a cocktail of MSNs of varying pore sizes, each one of which is optimized for one of the drugs in the combination.

### 3.2 Preparation and characterization of MSN-loaded DMAP

**Fig. 3:**
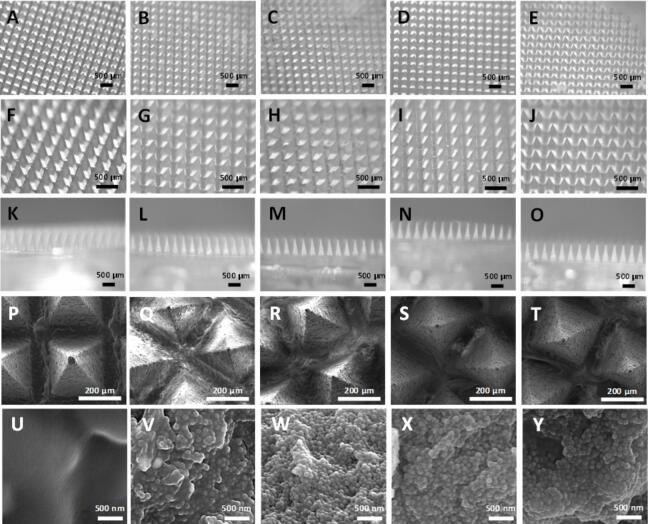
Stereomicroscopy (A-O) and SEM (P-Y) images of DMAP-containing MSNs at different magnifications and orientations. Blank DMAP without MSN (A,F,K,P,U); S-MSN (B,G,L,Q,V); M−MSN (C,H,M,R,W); L-MSN (D,I,N,S,X) and XL-MSN (E,J,O,T,Y).

DMAP-containing MSNs of different pore sizes were prepared using PVP and PVA (2 water-soluble polymers). Three different methods were evaluated to prepare PVP and PVA-based DMAP containing MSN: 1) dispersing MSN directly in the PVP-PVA mixture and removing excess after centrifugation before adding a second layer of PVP-PVA mixture without MSN; 2) Adding MSN in an aqueous suspension, removing excess after centrifugation and later adding PVP-PVA mixture and 3) filling the molds with powder MSN and later adding PVP-PVA mixture. It is worth noting that, when attempting the first method, we found that when large percentages of MSNs were included in the polymer mixture (>40 %), its viscosity was too high to homogeneously fill the mold used to obtain DMAP. As our aim was to prepare DMAP with a large amount of MSNs located in the microneedle tips, this fact led us to explore the other 2 alternative methods mentioned. DMAP containing MSN were successfully prepared by the 3 proposed methods, obtaining the following amounts of FITC-labelled M−MSN per DMAP: 4.01 ± 0.62 mg (method 1), 3.96 ± 0.39 mg (method 2) and 2.29 ± 0.06 mg (method 3). Importantly, as only the microneedle tips will be inserted inside the skin upon DMAP application, the MSNs should be selectively located in the microneedle tips to avoid unnecessary wastage of drug-loaded particles in future therapeutic applications of these formulations. To confirm the location of the MSNs within the patches, FITC-labelled M−MSNs were prepared and used to obtain DMAP. When comparing the distribution of the MSN throughout the microneedles (Figure S3), only the DMAP prepared by method 3 (filling the mold with MSN powder and later adding the PVP-PVA solution) presented selective location of the MSN at the microneedle tips. For this reason, as only the nanoparticles that are at the microneedle tips will be successfully deposited inside the skin (due to incomplete insertion), this method was selected for the rest of the experiments. Thus, DMAP with MSN of different pore sizes were prepared using this method. The stereomicroscopy images of the obtained DMAP (Fig. 3) confirm the successful preparation of the desired arrays with different MSN types presenting well-defined microneedles of the expected length (*ca.* 750 µm) and with intact tips. No morphological differences were found between the blank DMAP (without nanoparticles) and those containing the different types of MSNs. Furthermore, the SEM micrographs (Fig. 3**P-Y**) confirm the presence of MSNs, which make up most of the microneedle tips, as seen in the higher magnification micrographs (Fig. 3**U-Y**). These results confirm the preparation of DMAP in which the majority of the microneedle tips are composed of MSNs, in contrast with previous reports, where the amount of MSNs included in DMAP formulations was relatively low (Zhang et al., 2023). Finally, two-photon fluorescence microscopy images obtained from these formulations (Figure S4) confirm the presence of the MSNs only in the microneedle tips.

### 3.3 *In vitro* and *ex vivo* evaluation of MSN-loaded DMAP

**Fig. 4:**
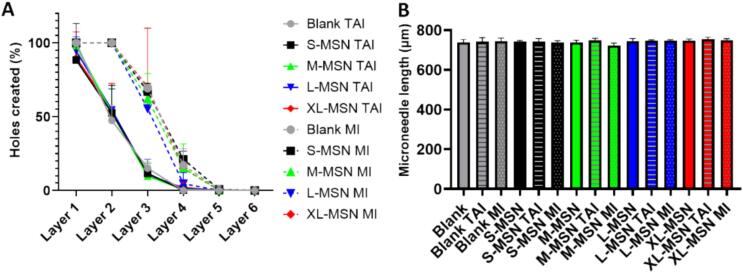
DMAP insertion in the Parafilm M® model either using a Texture Analyzer (TAI) (continuous lines) or through manual insertion (MI) (dotted lines) (A). Blank (not containing MSNs) or MSN-containing DMAP were evaluated. Microneedle lengths before and after insertion in the Parafilm M® model (B). Data are means ± SDs, n = 3.

The mechanical properties of the prepared DMAP and whether they can be inserted into the skin were first evaluated *in vitro* using a previously reported Parafilm M® model(Larrañeta et al., 2014). DMAP insertion was evaluated either using a Texture Analyzer that applied 32 N for 30 s or by manual application of thumb pressure for 30 s. The results (Fig. 4) show that the insertion of all the prepared DMAP was similar when inserted in the same way, regardless of whether the DMAP contained any kind of MSN. Furthermore, the DMAPs were inserted more efficiently by manual application (successfully piercing through the 3rd Parafilm M® layer, with a depth of 378 µm) compared to the ones inserted using the Texture Analyzer equipment (which only reached the 2nd layer, at a depth of 252 µm). These results are in good agreement with previous reports of microneedle patches that show similar insertion in the Parafilm M® model, as well as deeper insertion upon manual force (Courtenay et al., 2020). Then, the mechanical properties of the prepared DMAP were also proven to be adequate for further evaluation, as there was no significant change (p > 0.54) in microneedle length after insertion in the Parafilm M® model, neither by manual nor Texture Analyzer application (Fig. 4**B**). Further mechanical evaluation was carried out by applying 32 N for 30 s against the metal plate of the Texture Analyzer and evaluating the % of height reduction. The results (Figure S5) revealed an improvement in the mechanical properties of the DMAP by the introduction of MSN (regardless of pore size), as the % height reduction was significantly lower than that of the blank DMAP. The *in vitro* dissolution of the different DMAP formulations in aqueous media was also confirmed by immersing them in PBS. Three minutes after immersion, the microneedles of all DMAP were fully dissolved, as observed by stereomicroscopy (Figure S6). By dissolving in PBS DMAP containing FITC-labelled M−MSNs and then measuring the fluorescence of the resulting suspensions, the amount of nanoparticles loaded in each DMAP could be estimated to be 2.33 ± 0.04 mg M−MSNs/DMAP.

**Fig. 5:**
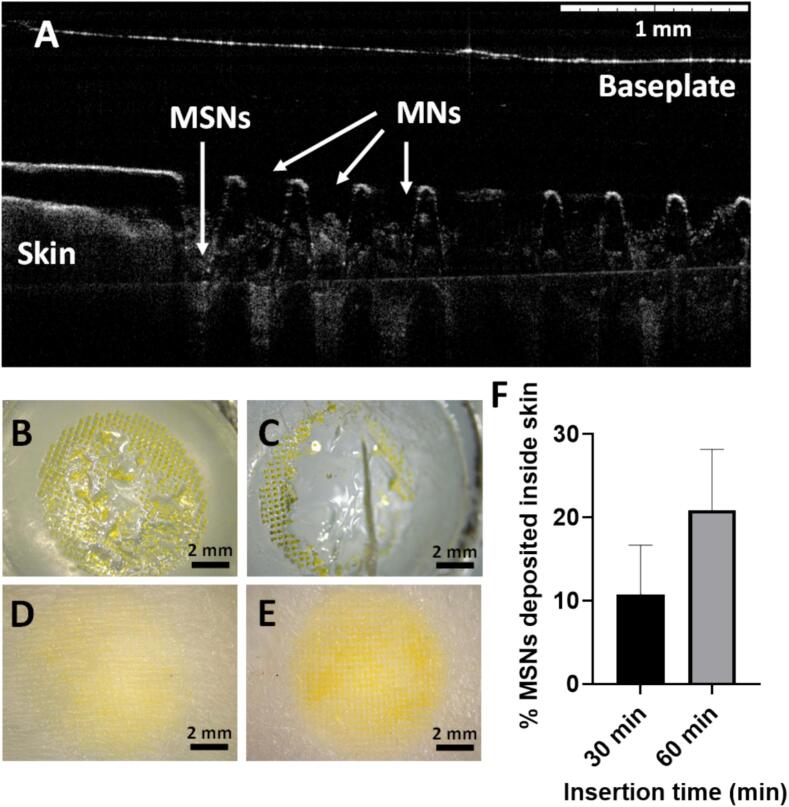
DMAP insertion and dissolution *ex vivo* using neonatal porcine skin. OCT image of M−MSN−containing DMAP inserted in neonatal porcine skin (A). Photographs of FITC-labelled M−MSN−containing DMAP after insertion in skin for 30 (B) or 60 (C) min. Photographs of neonatal porcine skin after removal of FITC-labelled M−MSN−containing DMAP that had been inserted for 30 (D) or 60 (E) min. Quantification of FITC-labelled M−MSN deposited inside neonatal porcine skin at different insertion times (F). Data are means ± SDs, n = 3.

The next step was to evaluate the insertion and dissolution of DMAP in neonatal porcine skin, which will provide more relevant information towards the potential use of these formulations in humans. Porcine skin has been reported to be anatomically and histologically similar to human skin, and thus it can be used as a relevant surrogate model for penetration and permeability studies (Barbero and Frasch, 2009, Garland et al., 2012, Kong and Bhargava, 2011). After manual *ex vivo* application in porcine skin, the OCT images confirmed the successful insertion of the microneedles inside the porcine skin, with an average insertion length of 472 ± 26.7 µm (62.9 ± 3.5 % of the total microneedle length) (Fig. 5). Furthermore, the successful dissolution of the microneedles inserted in the skin was also observed, with partial dissolution taking place in DMAP inserted for 30 min at 37 °C and reaching almost complete dissolution at 60 min (Fig. 5B-C). At this time (60 min), the amount of FITC-labelled M−MSNs that had been deposited in the skin was 0.49 ± 0.17 mg, which was 20.9 ± 7.26 % of the total amount present in the DMAP (Fig. 5D-F).

**Fig. 6:**
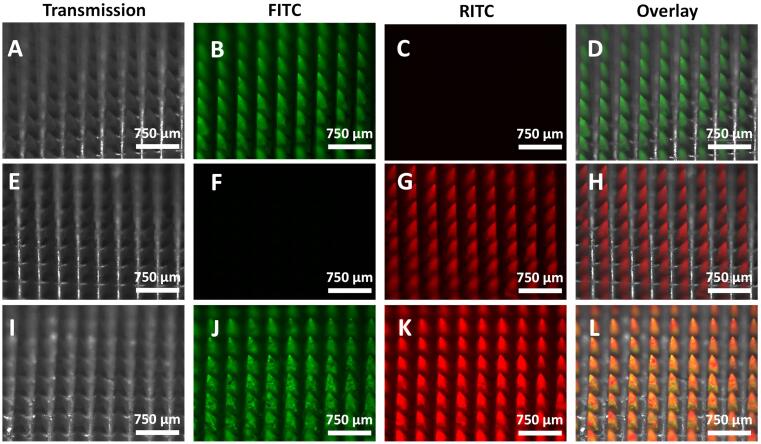
Fluorescence microscopy images of DMAP containing FITC-labelled M−MSNs (A-D), RITC-labelled XL-MSNs (E-H) or a combination of both types of MSNs (I-L).

As seen in the results shown in Fig. 2, cargo loading and release in MSNs is strongly affected by the interaction between cargo molecular weight and the pore size of the nanoparticles. For this reason, we postulate the potential use of combinations of MSNs of various pore sizes for combined drug codelivery, where each type of MSN is optimized for one drug in the mix. With this goal in mind, the next step was to evaluate whether the method developed to prepare DMAP would enable patches with combinations of different MSNs to be obtained. To evaluate this, we selected MSNs that presented optimal loading for each of the model molecules previously used: M−MSNs (which presented the largest fluorescein sodium salt loading capacity) and XL-MSNs (which presented the largest OVA loading capacity). To visualize each type of particle independently, we prepared and used differently labelled MSNs: FITC-labelled M−MSNs and RITC-labelled XL-MSNs. After mixing (in suspension) both types of MSNs in a 1:1 wt ratio and drying the combination, the MSN mix was used to prepare DMAP following the same method as previously described. The characterization of these new DMAPs confirms the successful preparation of DMAPs containing a combination of MSNs of different pore sizes (Fig. 6, Figure S7**,** Table S2). Furthermore, DMAP containing a combination of FITC-labelled M−MSNs and RITC-labelled XL-MSNs presented analogous insertion and nanoparticle deposition in neonatal porcine skin as those previously evaluated containing only one MSN type (Figure S8, Table S3).

**Fig. 7:**
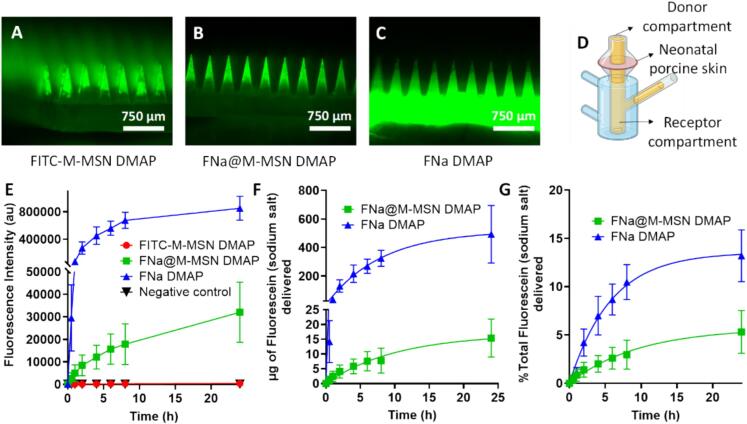
Franz cell experiment with neonatal porcine skin. Fluorescence microscopy images of the different DMAPs used in the experiment: FITC-labelled M−MSNs (FITC-M−MSN DMAP) (A), fluorescein sodium salt-loaded M−MSNs (FNa@M−MSN DMAP) (B), and nonencapsulated fluorescein sodium salt (FNa DMAP) (C). Schematic representation of the Franz cell setup (D). Fluorescence intensity in the receptor compartment (indicating transdermal delivery) at different time points after DMAP insertion in neonatal porcine skin (E). Amount (in µg) of total fluorescein sodium salt delivered transdermally at different time points (F). Percentage of total fluorescein sodium salt delivered transdermally at different time points (G). Data are means ± SDs, n = 5.

In a potential future application of these DMAPs, after insertion and microneedle dissolution, drug-loaded MSNs will be deposited inside the skin. The fate of both MSNs and their cargo after deposition in the skin should therefore also be evaluated to determine the potential of these formulations. To evaluate the diffusion of both the nanoparticles and their cargo, DMAP with 2 different nanoparticle systems was prepared: FITC-labelled M−MSNs (to analyse the fate of the nanoparticles) and fluorescein sodium salt-loaded M−MSNs (to analyse the fate of the fluorescent cargo as a model for a drug being released from the nanoparticles). In the first experiment, an agarose gel was used as a model for skin tissue. After insertion of DMAP, the microneedles dissolve and leave the MSNs inside the agarose gel. One hour later, sections of the gel were cut and evaluated by fluorescence microscopy. The results (Figure S9) show that while the MSNs remain in the site of deposition, the cargo (fluorescein sodium salt) diffuses out of the nanoparticles and into the gel. This would indicate that the nanoparticles would remain in the site of insertion, acting as a depot system and providing a sustained release of the drug to the surrounding skin tissue. To confirm these results, a second experiment was carried out in a Franz cell setup. The same DMAP as those from the previous experiment (plus an additional control DMAP with nonencapsulated fluorescein sodium salt) were inserted in neonatal porcine skin that acted as the membrane separating the donor and receptor compartments. At different time points, samples were taken from the receptor compartment, and the amount of FITC-labelled M−MSNs or free fluorescein sodium salt was evaluated by fluorimetry. The results (Fig. 7) confirm those from the previous experiment, showing that MSNs are not capable of diffusing through the skin tissue, as no nanoparticle fluorescence was detected in the receptor compartment. On the other hand, fluorescein sodium salt crosses through the skin tissue after being deposited with DMAP. Importantly, encapsulation of the dye inside M−MSNs provided sustained release compared to the use of DMAP with a nonencapsulated fluorophore. Taken together, these results indicate that the MSNs deposited in the skin through DMAP remain at the site of insertion and act as a drug reservoir. Then, the sustained release of the drug would provide a continuous flow of the drug towards both the surrounding tissue or even systemic circulation (depending on the release kinetics and dose of the drug or nanoparticles included in the patches).

### 3.4 *In vivo* evaluation of MSN-loaded DMAP

**Fig. 8:**
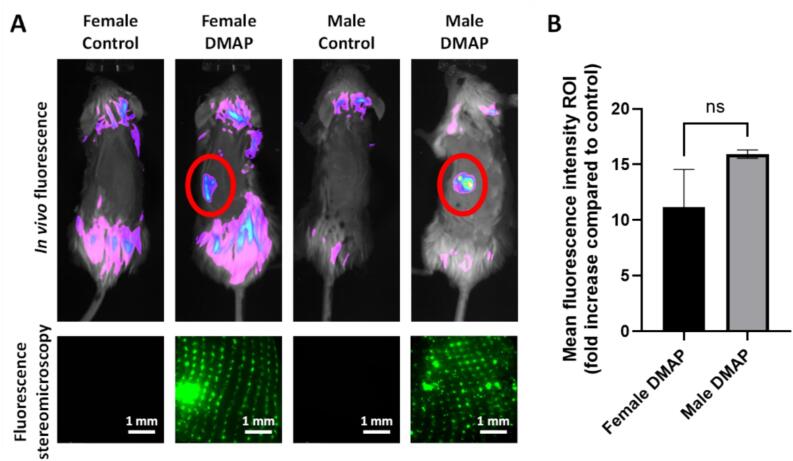
DMAP insertion, dissolution and MSN deposition *in vivo*. *In vivo* fluorescence imaging of both controls and FITC-labelled M−MSN−loaded DMAP-inserted male and female mice taken 3 days after DMAP administration (A, top). *Ex vivo* fluorescence stereomicroscopy of skin excised from the same mice showing fluorescence of the deposited FITC-labelled M−MSNs (A, bottom). Fold increase in mean fluorescence intensity at the insertion area compared to control for both female and male mice (quantified from the *in vivo* fluorescence images), showing no significant difference among them (B). Data are Means ± SD, n = 3.

Once the characteristics and performance of MSN-loaded DMAP had been evaluated through the different *in vitro* and *ex vivo* experiments described above, a series of *in vivo* tests were carried out using a mouse model to examine the therapeutic potential of the developed platform. First, the *in vivo* insertion, microneedle dissolution and nanoparticle deposition were evaluated by a combination of *in vivo* fluorescence and *ex vivo* fluorescence stereomicroscopy (Fig. 8). The results confirm the successful insertion, microneedle dissolution and nanoparticle deposition, as nanoparticle fluorescence is clearly visible in both the *in vivo* fluorescence imaging and the *ex vivo* fluorescence stereomicroscopy images 3 days after DMAP administration to the mice. The insertion of the DMAP was successful in both male and female mice, requiring 2 h of insertion for almost complete microneedle dissolution in the skin (with only partial dissolution after 1 h). No significant differences were found in the mean *in vivo* fluorescence intensity at the microneedle insertion site between male and female mice (Fig. 8B). In the *ex vivo* fluorescence stereomicroscopy images, the microneedle pattern is clearly visible in the green fluorescence, confirming successful nanoparticle deposition in DMAP-treated mice (both male and female), while no fluorescence was observed in the skin of control animals. The quantification of the total amount of deposited FITC-labelled M−MSNs (carried out using the same method as described for the *ex vivo* experiments in neonatal porcine skin) provided an estimation of 134.2 ± 79.3 µg of MSNs deposited inside the mouse skin per DMAP application.

**Fig. 9:**
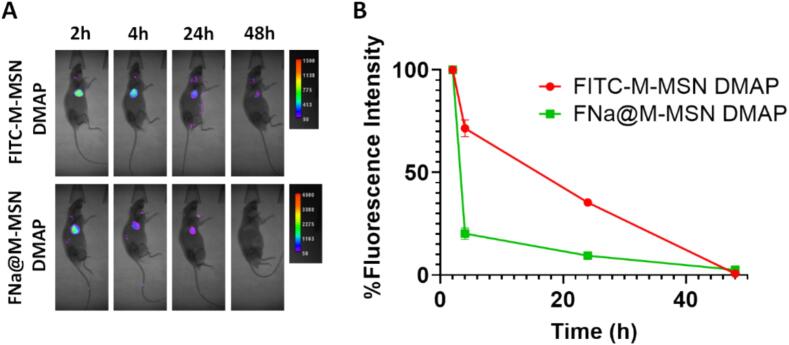
*In vivo* fluorescence imaging of mice at different times after insertion of FITC-M−MSN DMAP or FNa@M−MSN DMAP (A); Quantification of Fluorescence intensity in the area where DMAP had been inserted at different timepoints (B).

Furthermore, cargo release from deposited MSN was confirmed *in vivo* by evaluating the *in vivo* fluorescence over time after insertion of DMAP containing FITC-labelled M−MSNs (to follow nanoparticle location) and fluorescein sodium salt-loaded M−MSNs (to follow cargo location). The results (Fig. 9) show a slow decrease of nanoparticle fluorescence in the insertion area, which indicates a relatively slow disappearance of the nanoparticles from the skin, either due to removal by cells or by degradation and dissolution of the silica structure. On the other hand, cargo fluorescence decreased very rapidly in the first hours after insertion (with around 80 % decrease in fluorescence between 2 and 4 h after insertion). These results are in good agreement with the fast release kinetics of fluorescein (sodium salt) from MSNs and confirm the successful release of the cargo *in vivo* after nanoparticle deposition in the skin.

## 4 Conclusions

In this work, a new nanoparticle powder-based methodology was demonstrated to prepare dissolving microneedle array patches containing large amounts of mesoporous silica nanoparticles of different pore sizes located mainly at the microneedle tips. The successful insertion, dissolution and nanoparticle deposition from the microneedles as well as the subsequent cargo release from the nanoparticles were confirmed through a series of *in vitro*, *ex vivo* and *in vivo* experiments. Thus, the modular methodology described here enables the easy and simultaneous incorporation of different drug release nanoparticles in dissolving microneedle array patches that could be used for combined therapy in various clinical scenarios. Furthermore, compared to previous work, the use of dry MSN powder for the preparation of the microneedles streamlines the process, as this dry form will be more stable, enabling the preparation of a stock of nanoparticles loaded with different drugs that can be stored until needed for the preparation of microneedle patches, preventing drug or nanoparticle degradation as well as undesired premature release to the liquid medium. The use of powder nanoparticles also reduces nanoparticle waste during the preparation of microneedles, as is common when preparing suspensions of nanoparticles in polymer solutions (in excess) that will be added to the molds. This work presented the evaluation of the prepared microneedle arrays *ex vivo* in neonatal porcine skin and *in vivo* using mice. While pig skin is commonly used as a model for human skin, the evaluation of this system in human skin would still be desirable in the future to assess its potential for human therapeutics, as well as its *in vivo* evaluation using larger animals, before considering clinical testing. Further work will also be needed to evaluate this modular platform for specific therapeutic applications using MSN loaded with specific drugs tailored to each potential application.

## CRediT authorship contribution statement

**Juan L. Paris:** Writing – original draft, Methodology, Investigation, Funding acquisition, Formal analysis, Data curation, Conceptualization. **Lalitkumar K. Vora:** Writing – review & editing, Supervision, Methodology, Formal analysis, Conceptualization. **Ana M. Pérez-Moreno:** Writing – review & editing, Methodology, Investigation. **María del Carmen Martín-Astorga:** Writing – review & editing, Investigation. **Yara A. Naser:** Writing – review & editing, Methodology, Investigation. **Qonita Kurnia Anjani:** Writing – review & editing, Methodology, Investigation. **José Antonio Cañas:** Writing – review & editing, Methodology, Investigation. **María José Torres:** Writing – review & editing, Supervision, Methodology, Investigation. **Cristobalina Mayorga:** Writing – review & editing, Supervision, Project administration, Methodology, Funding acquisition, Conceptualization. **Ryan F. Donnelly:** Writing – review & editing, Supervision, Resources, Project administration, Methodology, Funding acquisition, Formal analysis, Conceptualization.

## Declaration of competing interest

The authors declare the following financial interests/personal relationships which may be considered as potential competing interests: Cristobalina Mayorga reports financial support was provided by Carlos III Health Institute. Juan L Paris reports financial support was provided by State Agency of Research. Ryan F. Donnelly reports financial support was provided by Wellcome Trust. Juan L Paris reports financial support was provided by Carlos III Health Institute. Jose A. Canas reports financial support was provided by Carlos III Health Institute. Cristobalina Mayorga reports financial support was provided by Regional Government of Andalusia Ministry of Health and Consumer Affairs. Prof. Ryan F. Donnelly is part of the Editorial Board of International Journal of Pharmaceutics. If there are other authors, they declare that they have no known competing financial interests or personal relationships that could have appeared to influence the work reported in this paper.

## Data Availability

Data will be made available on request.
