# Supplementary material for: Dissolving microneedle array patches containing mesoporous silica nanoparticles of different pore sizes as a tunable sustained release platform
Source: Int J Pharm. 2025 Jan 25;669:125064. doi: 10.1016/j.ijpharm.2024.125064 (PMC11757158; doi:10.1016/j.ijpharm.2024.125064)
Supplement: Supplementary Data 1 [file mmc1.pdf]

SUPPORTING INFORMATION

FOR

Dissolving microneedle array patches containing mesoporous silica nanoparticles of different pore sizes as a tunable sustained release platform

Juan L. Paris[1,3,*], Lalitkumar K. Vora[3], Ana M. Pérez-Moreno[1,2], María del Carmen Martín-Astorga[1,2], Yara A. Naser[3], Qonita Kurnia Anjani[3], José Antonio Cañas[1], María José Torres[1,2,4], Cristobalina Mayorga[1,4,*], Ryan F. Donnelly[3,*]

[1]Allergy Research Group, Instituto de Investigación Biomédica de Málaga y Plataforma en Nanomedicina-IBIMA Plataforma BIONAND. RICORS "Enfermedades inflamatorias", Málaga, Spain;
[2]Universidad de Málaga, Málaga, Spain.
[3]School of Pharmacy, Queen's University Belfast, Medical Biology Centre, Belfast, Northern Ireland, UK;
[4]Allergy Unit, Hospital Regional Universitario de Málaga-HRUM, Málaga, Spain
Corresponding authors e-mail: juan.paris@ibima.eu; lina.mayorga@ibima.eu; r.donnelly@qub.ac.uk

Table S1. Loading Capacity (LC, w/w %) and Encapsulation Efficiency (EE, %) of Fluorescein (sodium salt), insulin and OVA in MSN of different pore sizes.

	**Fluorescein (sodium salt)**		**Insulin**		**Ovalbumin**	
	LC (%)	EE (%)	LC (%)	EE (%)	LC (%)	EE (%)
S-MSN	24.3 ± 0.6	32.0 ± 1.1	10.4 ± 0.1	90.3 ± 0.9	16.9 ± 0.3	20.4 ± 0.4
M-MSN	25.9 ± 0.6	35.1 ± 1.1	11.2 ± 0.3	98.1 ± 3.1	19.0 ± 0.5	23.5 ± 0.7
L-MSN	9.0 ± 1.8	9.9 ± 2.2	11.3 ± 0.1	99.8 ± 0.2	21.8 ± 0.3	27.9 ± 0.4
XL-MSN	8.3 ± 4.5	9.2 ± 5.4	11.3 ± 0.1	99.5 ± 0.4	25.9 ± 0.1	34.9 ± 0.2

Table S2. Characterization of the amount of fluorescently-labeled MSNs per DMAP.

	Amount of FITC-M-MSN per MNA (mg)	Amount of RITC-XL-MSN per MNA (mg)	Total amount of MSN per MNA (mg)
FITC-M-MSN DMAP	2.29 ± 0.06	N/A	2.29 ± 0.06
RITC-XL-MSN DMAP	N/A	2.31 ± 0.41	2.31 ± 0.41
FITC-M-MSN + RITC-XL-MSN DMAP	0.88 ± 0.05	1.56 ± 0.07	2.43 ± 0.08

Table S3. Evaluation of nanoparticle deposition of fluorescently-labeled MSNs per DMAP.

	FITC-M-MSN Deposition %	RITC-XL-MSN Deposition %	Total MSN Deposition %
FITC-M-MSN DMAP	20.88 ± 7.26	N/A	20.88 ± 7.26
RITC-XL-MSN DMAP	N/A	24.01 ± 14.02	24.01 ± 14.02
FITC-M-MSN + RITC-XL-MSN DMAP	21.184 ± 10.68	23.50 ± 9.23	22.72 ± 8.51

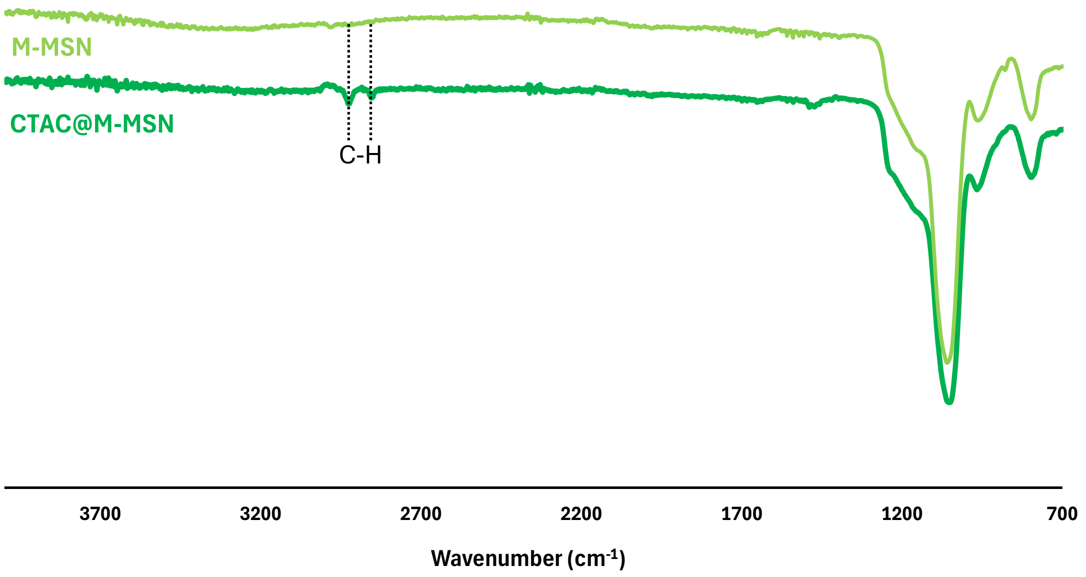

Figure S1. Representative FTIR spectra of M-MSN before (CTAC@M-MSN) and after (M-MSN) surfactant extraction confirming the successful removal of the surfactant through the disappearance of C-H bands in the extracted material.

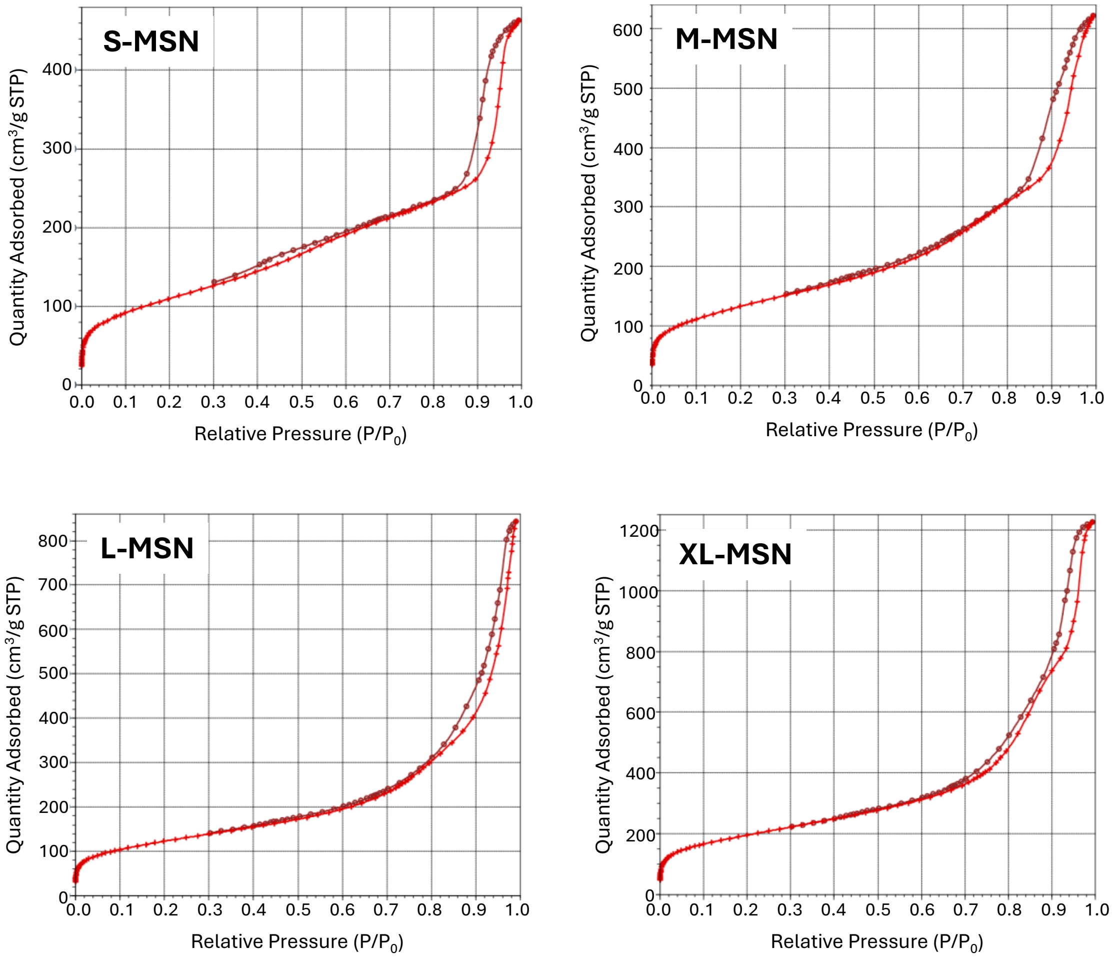

Figure S2. N_2 adsorption/desorption isotherms of the prepared MSN.

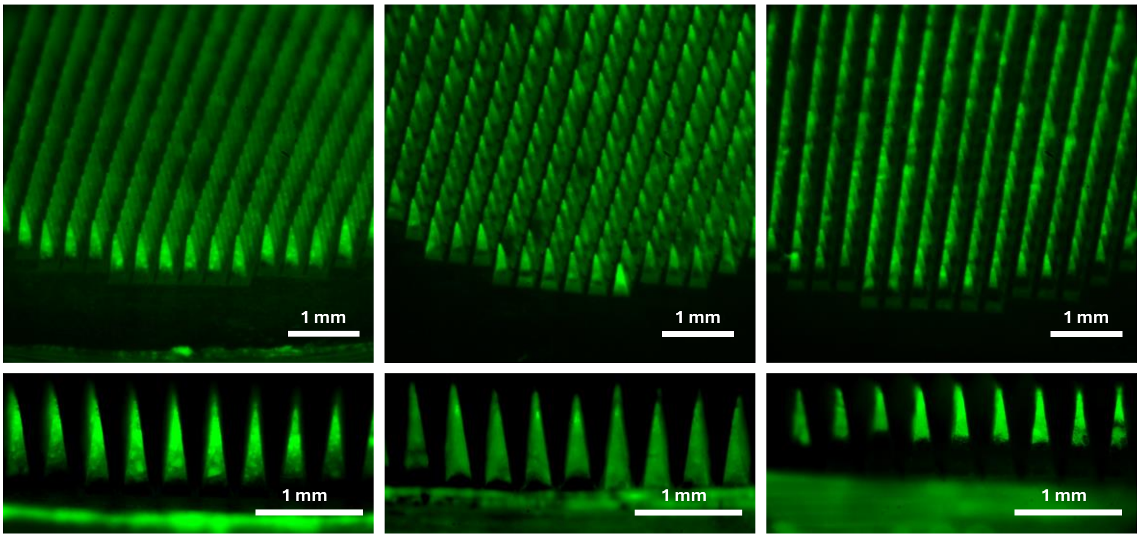

Figure S3. Fluorescence stereomicroscopy images of DMAP with FITC-Labeled M-MSN prepared by 3 different methods: i) dispersing MSN directly in the PVP-PVA mixture and removing excess after centrifugation before adding a second layer of PVP-PVA mixture without MSN (left); ii) Adding MSN in an aqueous suspension, removing excess after centrifugation and later adding PVP-PVA mixture (center) and iii) filling the molds with powder MSN and later adding PVP-PVA mixture (right).

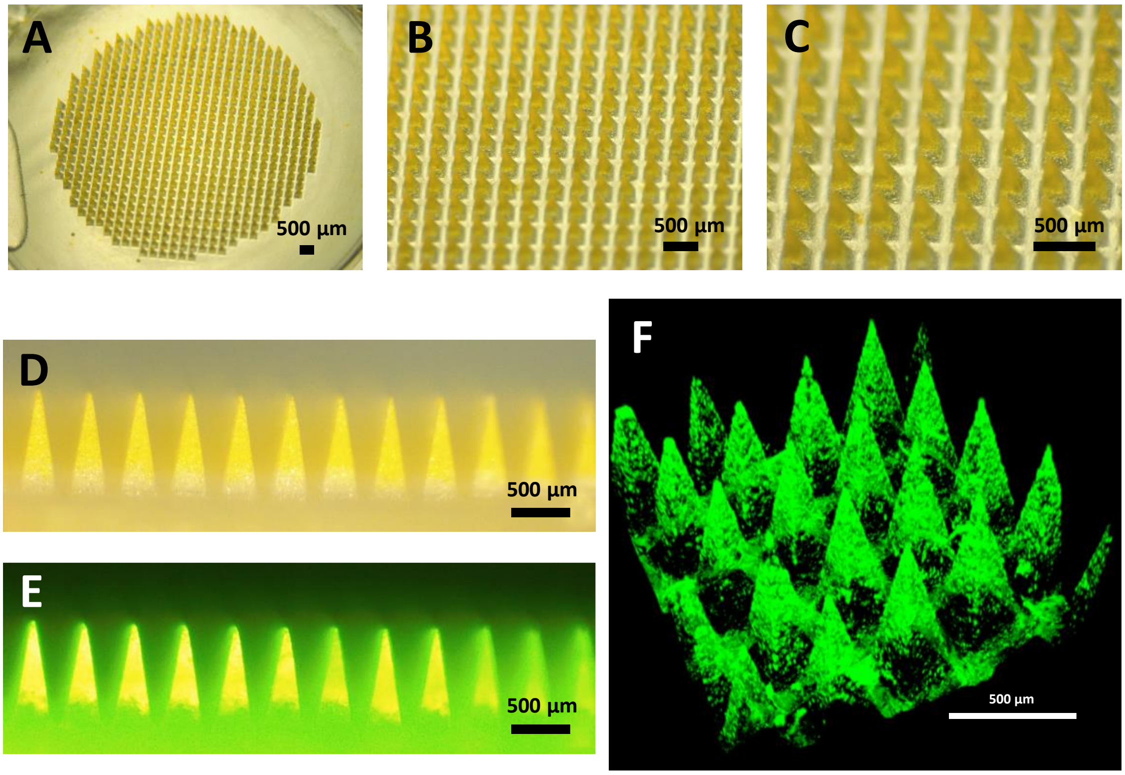

Figure S4. DMAP containing FITC-labeled M-MSN. Stereomicroscopy images (A-D), Fluorescence stereomicroscopy (E) and 3D reconstruction of DMAP using Two-photon fluorescence microscopy, all showing selective location of the nanoparticles in the microneedle tips.

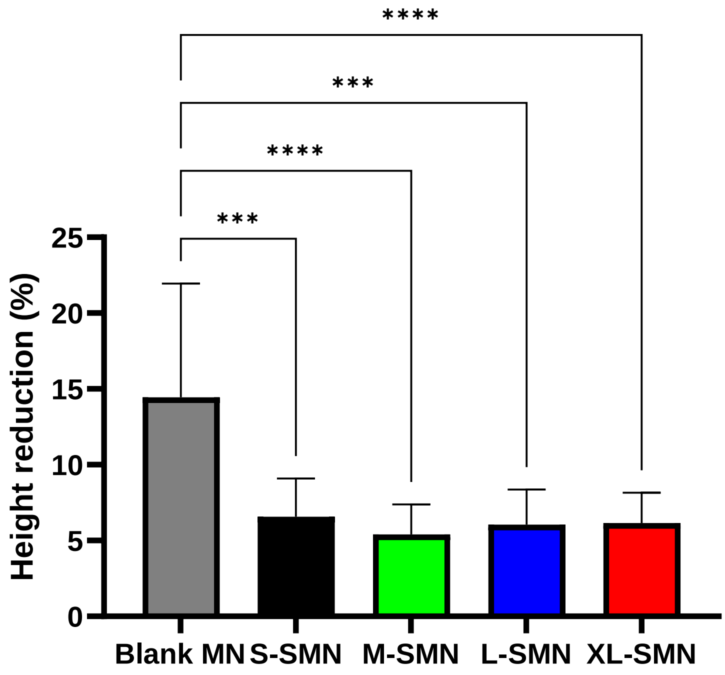

Figure S5. Percentage of height reduction in the different DMAP formulations after application of 32 N for 30 seconds against a metal plate.

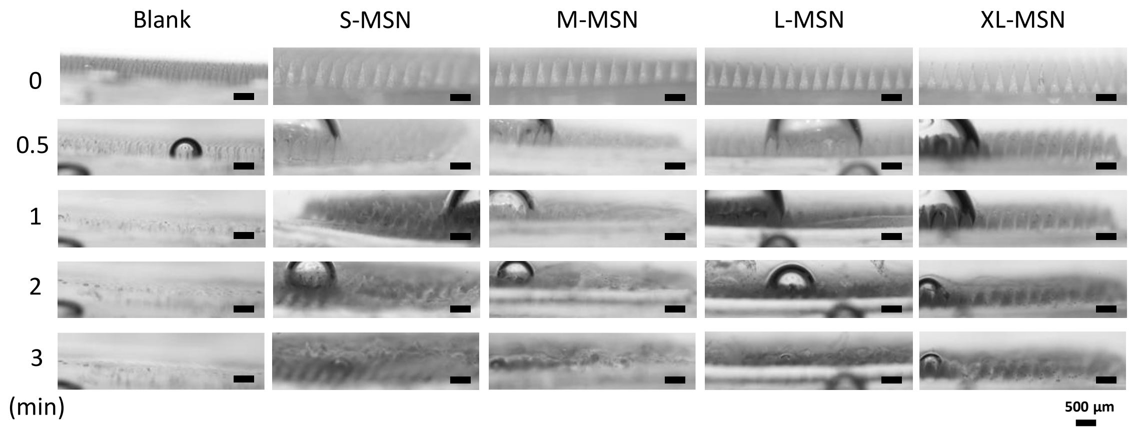

Figure S6. Stereomicroscopy images showing the *in vitro* dissolution of the different DMAP formulations after up to 3 min immersed in PBS.

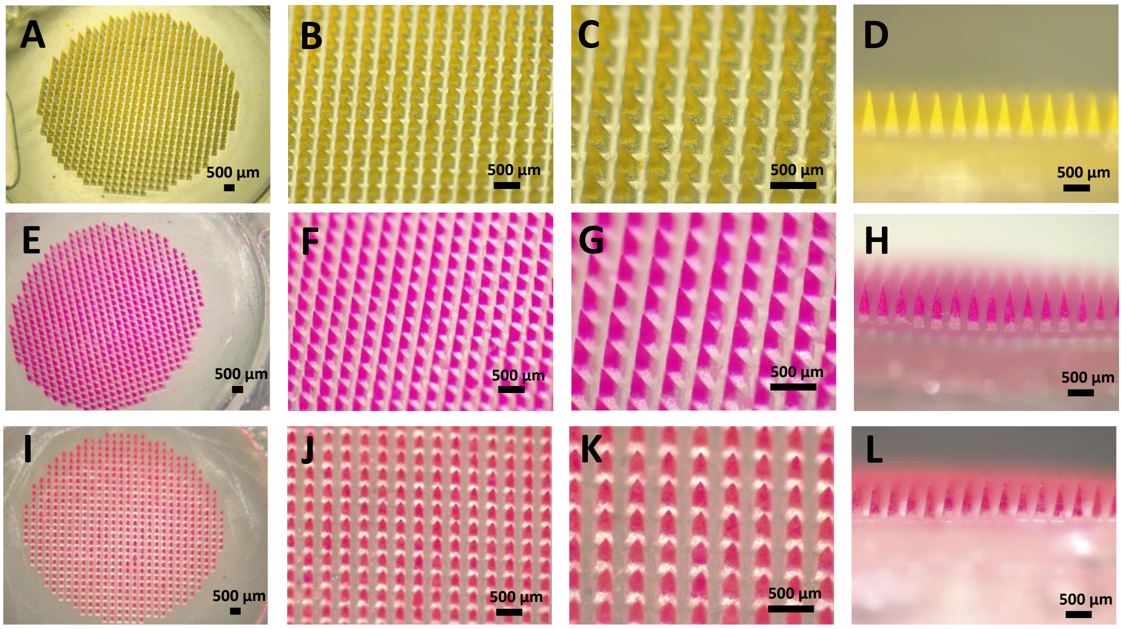

Figure S7. Stereomicroscopy images of DMAP containing FITC-labeled MSNs (A-D), RITC-labeled XL-MSN (E-H) or a combination of both types of MSNs (I-L).

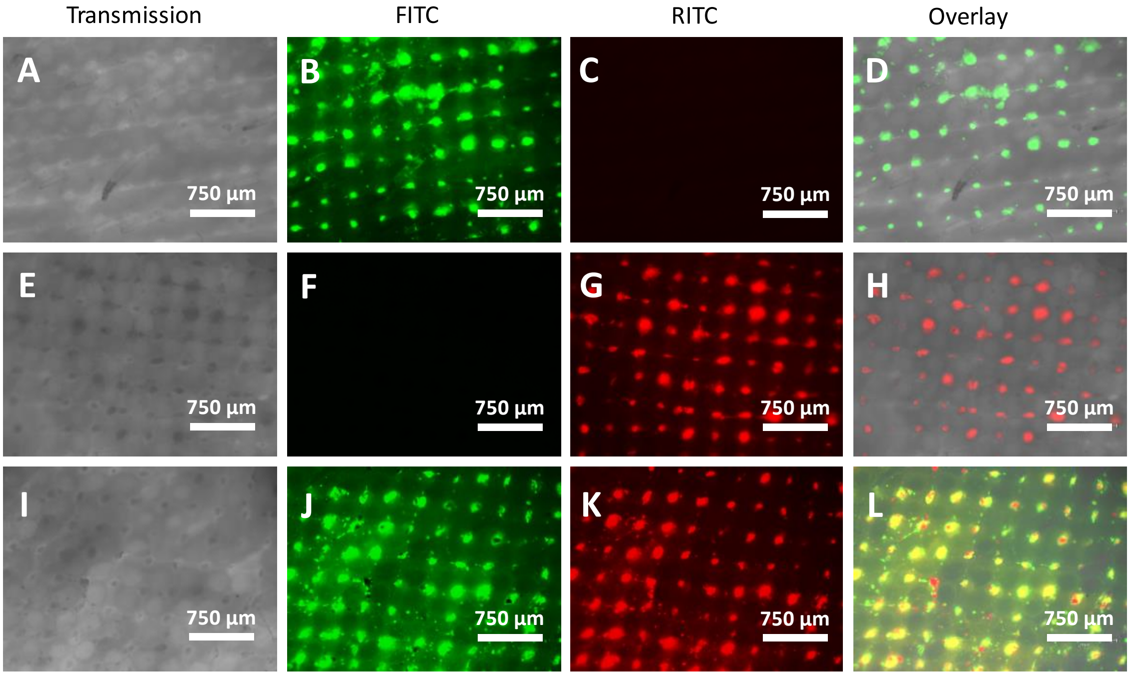

Figure S8. Fluorescence microscopy images of neonatal porcine skin after removal of DMAP containing FITC-labeled MSNs (A-D), RITC-labeled XL-MSN (E-H) or a combination of both types of MSNs (I-L).

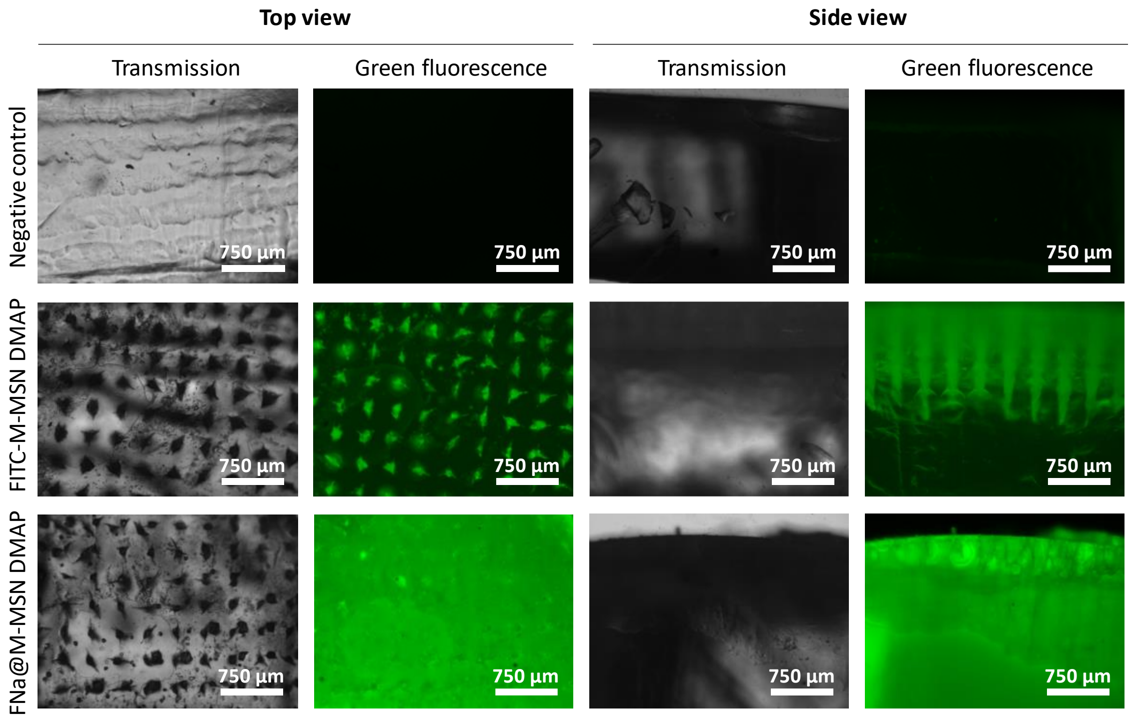

Figure S9. Fluorescence microscopy images of agarose gels 1 hour after insertion of DMAP without nanoparticles (top), containing FITC-labeled MSNs (center) and containing fluorescein sodium salt-loaded M-MSNs (bottom).
